# Decoding of Inconsistent Biological Data: A Critical Step toward Enhanced AI Predictivity in Drug Discovery

**DOI:** 10.1021/acsptsci.5c00677

**Published:** 2025-12-14

**Authors:** Mira A. M. Behnam, Andrea Cavalli, Diana Lousa, Cláudio M. Soares, Christian D. Klein

**Affiliations:** † Medicinal Chemistry, Institute of Pharmacy and Molecular Biotechnology, Heidelberg University, Im Neuenheimer Feld 364, Heidelberg 69120, Germany; ‡ 27218 Centre Européen de Calcul Atomique et Moléculaire (CECAM), Ecole Polytechnique Fédérale de Lausanne, Lausanne 1015, Switzerland; § 98819 Instituto de Tecnologia Química e Biológica António Xavier, Universidade Nova de Lisboa, Av. da República, Oeiras 2780-157, Portugal

**Keywords:** Artificial intelligence, machine learning, bioactivity data, testing conditions, conformational plasticity, protease

## Abstract

Combining bioactivity data of assays against the same target, which are obtained from different sources, was recently shown to lead to considerable noise for training data sets of machine learning (ML) models. In this Viewpoint, we address the profound impact originating from often overlooked changes to an assay protocol relating to the buffer composition and experimental setup. We cover two examples of protein targets that undergo conformational changes driven by extrinsic factors: enzymes as catalytically active proteins, and viral surface proteins as structural targets. We discuss strategies to tackle this challenge for the case of enzyme inhibitors/binders, the utility of models based on deep learning (DL), and current limitations of computational studies assessing protein–ligand interactions. In an interview with an expert in the field of large language models (LLMs) and agentic AI, we explore how the latest developments in these areas can be leveraged to support drug discovery efforts.

## Graphical abstract

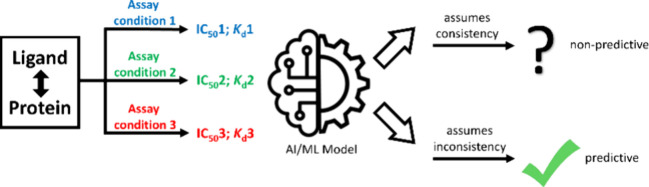

The need for sufficiently large data sets is considered a prerequisite for training and building artificial intelligence and machine learning (AI/ML) models to achieve good predictivity. This has prompted researchers to use expanded data sets, sometimes by merging heterogeneous and/or poorly curated data. For compound potency predictions, enlarging a data set by combining heterogeneous, nonconsistent results of biochemical or pharmacological assays can strongly limit the performance of the generated models.^1^ This problematic practice of compiling literature data from different sources was recently addressed in a study that provided a realistic example of the complexity of handling bioactivity results.^1^ By relying on publicly accessible assay-related information, listed in the ChEMBL database,^2^ the authors reported that even with their maximal curation strategy, the noise in the IC_50_ or *K*_i_ data could not be eliminated.^1^ In this Viewpoint, we address the profound impact of testing conditions on the inhibition and affinity values of compounds with examples from our work for assays conducted on enzymes, as isolated protein targets. Implementing strategies to mitigate this phenomenon adds yet another struggle for the development of AI/ML models, but can drastically improve the comparability of bioactivity data. To further demonstrate the impact of experimental variables on protein structure, we discuss the case of viral surface proteins that undergo major conformational rearrangement driven by pH. Several implications arise: Reference compounds that are used as points of comparison for different assay procedures should be carefully selected to reach the desired compatibility between heterogeneous data sets. Furthermore, testing conditions should be assessed relative to cellular or higher-order phenotypic systems (e.g., animal studies) to select the most relevant experimental setup(s) for the studied target. In particular, protein targets that exhibit conformational plasticity and therefore not a single fixed conformation but rather a “landscape” of conformational states, whose distribution varies based on extrinsic factors besides the ligand, will require dedicated efforts to enhance AI/ML model performance and predictivity. Data related to more than one experimental setup or target can be handled by clustering several ML models.

## Results and Discussion

### Assay Compatibility Considerations

The risk of combining results from different sources without careful assessment is not just related to the generated noise but to the concern of ending up with a model that lacks scientific relevance with respect to the studied target. Testing approaches for ligands against a particular target vary in several aspects: (i) different discipline-based assays (e.g., biochemical, biophysical, cell-based, etc.), (ii) different techniques within the same “domain” (e.g., in biophysics: isothermal titration calorimetry (ITC), surface plasmon resonance (SPR), biolayer interferometry (BLI), etc.), (iii) different assay conditions for the same technique (e.g., buffer, ionic strength, pH, additives, temperature, incubation time, etc.), (iv) different ways to fit the data and calculate results. Generally, data curation steps are limited to matching the indexed assay-related parameters—the metadata—in public repositories. However, despite the efforts and continuous updates, a number of assay-related details are not mentioned in such databases, and accordingly are not taken into account. These include, for example, substrate identity and concentration, buffer composition, and experimental setup for the assays behind IC_50_ and *K*_i_ values, as noted in ChEMBL.^1,2^ As an alternative, the results of shared compounds between different assay sets are used to judge their comparability.^1,3^ The selection commonly relies only on the compound identity without topic-specific knowledge about its mechanism of action, which could provide an inaccurate indication since compounds are not equally sensitive to variations introduced to assay protocols. The considerations we raise in this Viewpoint require attention to details that are so far not accessible in public databases. Nevertheless, these aspects can provide an opportunity to improve the quality of data sets obtained from combined data.

#### A) Variation of IC_50_, and *K*_d_ or *K*_i_ Values

Assays on an isolated protein target, e.g. enzymes or structural proteins, represent a straightforward approach to assess compound activity in vitro. Their specificity to the studied target, amenability for high-throughput, and limited complexity often make them the first decision-making checkpoint in testing pipelines for drug discovery. We focus in the following discussion on the case of enzymes. For IC_50_ values, the identity and concentration of the used substrate represent important factors that influence the results.^4^ When not accounted for, these aspects can provide one explanation for the noise in combined IC_50_ data. Dissociation constants (*K*_d_ or *K*_i_), on the other hand, are not usually based on substrate-related parameters.^4–6^ However, both IC_50_ and dissociation constants (*K*_d_ or *K*_i_) are subject to substrate-independent variation based on the experimental procedures used to generate them.^7,8^ This is often related to the influence of factors such as pH, ionic strength, temperature, and buffer composition on the distribution of existing protein conformations in solution based on the conformational selection model.^7,9^ Accordingly, the result of the assays vary, since ligands may have different preferences for distinct protein conformations. There could be as well ligand-specific explanations in certain cases, or effects on the transient intermediate formed by the initial collision between the ligand and the protein before the formation of a stable complex, a state referred to as the protein–ligand encounter complex.^7^ Most importantly, as shown recently, some of the testing conditions used for a particular target may have very limited predictive value for cellular activity and hit identification.^8^ These should be experimentally identified and excluded to avoid biasing the models and to prevent the prediction of false hits.

In our work on the main protease (M^pro^) of SARS-CoV-2, we noted that the IC_50_ of boceprevir differed by 4-fold depending on the buffer composition, using an enzyme concentration equal to 500 nM.^10^ Variation of the inhibitor IC_50_ was also observed for the protease of dengue virus (DENV) to a more significant extent,^7,8^ as shown for compound B in Figure 1. In both cases, the substrate identity and concentration were kept the same. Analysis of X-ray structures for both proteases revealed high plasticity for the ligand-free and ligand-bound protein,^11,12^ which is further confirmed by in-solution NMR studies.^7,12,13^

**Figure 1:**
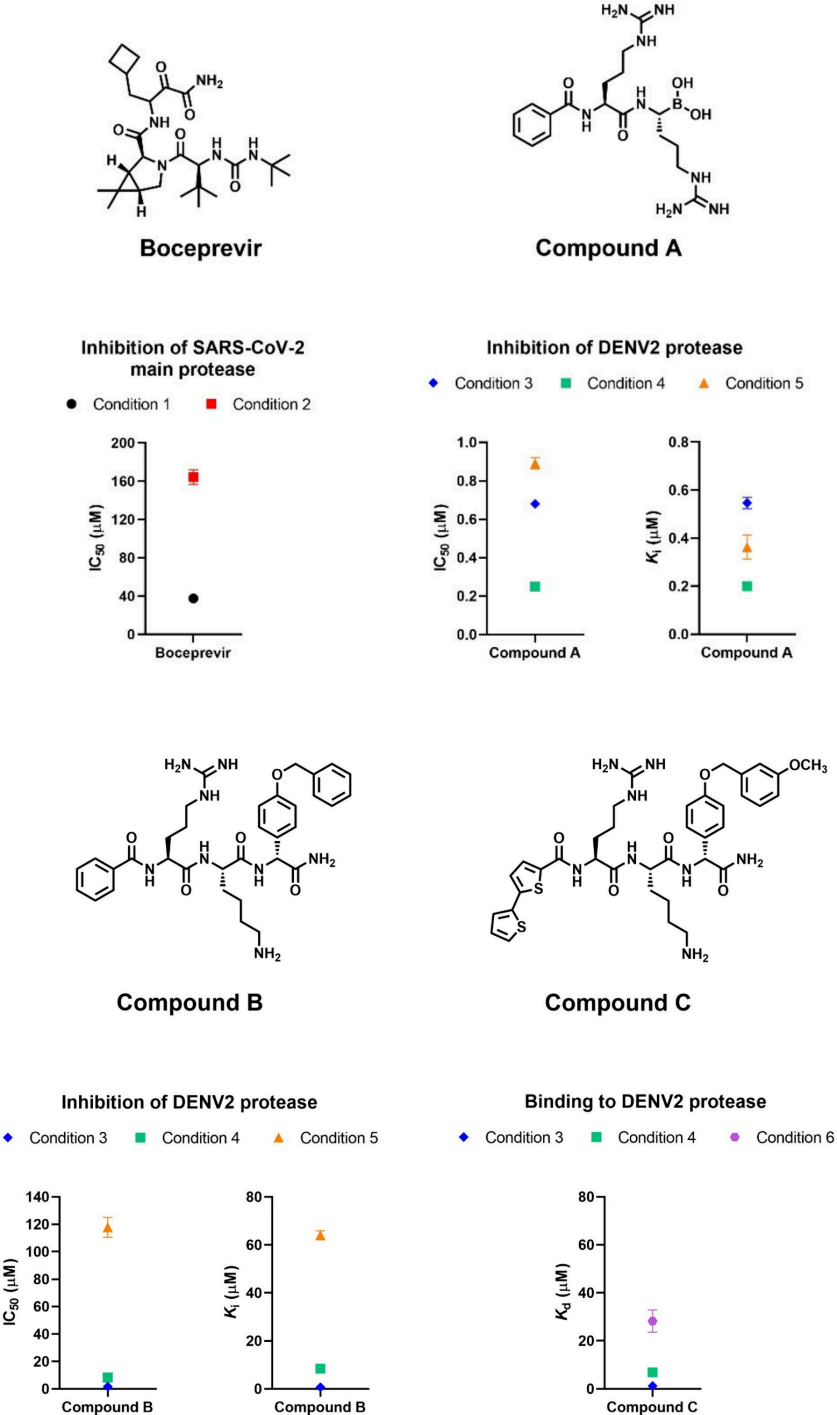
Substrate-independent variation of inhibition and binding depending on experimental conditions. The substrate identity and concentration were identical for the measured IC_50_ values. The substrate, Abz-VVTLQSGDap­(Dnp)­R-OH (2-aminobenzoic acid-valine-valine-threonine-leucine-glutamine-serine-glycine-2,4-dinitrophenyl-diaminopropionic acid-arginine-OH), at a concentration of 20 μM, was used for SARS-CoV-2 main protease, or the substrate, Bz-nKRR-AMC (benzoyl-norleucine-lysine-arginine-arginine-7-amino-4-methylcoumarin), at a concentration of 50 μM for DENV2 protease. For boceprevir, the plot is based on IC_50_ values determined in reference 10. For compounds A and B, the plots of IC_50_ values are based on results published in reference 7. *K*_i_ values were derived from IC_50_ values measured at different substrate concentrations, using the Cheng-Prussof plot, as previously described,^4,18^ due to the competitive nature of the inhibitors. *K*_d_ values were derived from the normalized tryptophan fluorescence of the protease determined in the presence of different concentrations of the 2,2′-bithiophene-containing ligand, as previously described.^5^ Results were analyzed using GraphPad Prism 8.0. Condition 1: His_6_-SARS-CoV-2 M^pro^, 40 mM HEPES buffer, pH 6.5, 20% glycerol, RT; condition 2: His_6_-SARS-CoV-2 M^pro^, 50 mM HEPES buffer, pH 6.5, 150 mM NaCl, RT; condition 3: His_6_-DENV2 pro, 50 mM Tris buffer, pH 9, 10% ethylene glycol, 1 mM CHAPS, RT; condition 4: His_6_-DENV2 pro, 50 mM Tris buffer, pH 7.5, 10% ethylene glycol, 1 mM CHAPS, RT; condition 5: nontagged DENV2 pro, 50 mM phosphate buffer, pH 7, 1 mM CHAPS, 37 °C; condition 6: nontagged DENV2 pro, 50 mM citrate buffer, pH 4, 1 mM CHAPS, 37 °C. The error bars for some points are small relative to the plot scale, so they cannot be seen in the figure. DENV2 stands for dengue virus serotype 2. RT stands for room temperature.

In the case of irreversible inhibitors, inhibition kinetics are particularly important. For this reason, the covalent inactivation efficiency constant, *K*_inact_/*K*_I_, is a better value to consider for ligands having this mechanism of action. *K*_inact_/*K*_I_ is a second-order rate constant that encompasses both the initial reversible or noncovalent binding event in the inactivation constant (*K*_I_) and the maximal rate of covalent inactivation (*K*_inact_).^14^

Another case we want to address is that of viral structural proteins, which are involved in the entry/fusion process, and that exhibit pH-driven conformational changes. This applies, for example, to the hemagglutinin protein of influenza viruses, the envelope protein (E protein) of orthoflaviviruses (e.g., tick-borne encephalitis virus, dengue virus, yellow fever virus, Zika virus, West Nile virus), and the glycoprotein complex (GPC) of Lassa virus.^15,16^ For these surface proteins, the acidic environment inside the endosome is essential for the fusion process; therefore, pH is a critical factor in binding assays. In cellular replication assays, temperature (4 °C or 37 °C) is used to distinguish between drugs interfering with virus binding/attachment events and entry/fusion events, respectively. The latter are characterized by highly dynamic interactions with transiently exposed binding surfaces.^17^

The most important consideration when choosing assay conditions to determine activity or binding parameters on an isolated protein target, e.g. enzymes or viral surface proteins, is the translational relevance. This means how the obtained results, and thus the assessed protein conformation(s), correlate with the outcomes of more complex cellular assays or in vivo models. In this regard, our work on the biochemical enzymatic assays of DENV2 protease revealed that optimization of assay conditions based only on the technical readout, i.e. the signal-to-noise ratio, of specific substrates can lead to protocols that have little resemblance to physiological conditions (e.g., Tris buffer, pH 9).^8^ Caution should be addressed to the choice of buffer additives, such as detergents, as they may influence protein conformation, and thus the identification of certain classes of inhibitors/binders.^7,8^ Detergents may also occupy binding sites that are relevant for inhibitor recognition and therefore have a direct interference with ligand binding. A famous example for this effect is the occupancy of a potentially druggable binding site in the E protein of orthoflaviviruses by the detergent *n*-octyl-β-d-glucoside (β-OG).^19^ Instead, conditions identified from large-scale correlations of IC_50_ and EC_50_ values offer a better approach for optimizing testing conditions. Specific protein conformational states may be more relevant than others for the development of therapeutics, as discussed previously for vaccine development based on the spike protein of SARS-CoV-2.^7^ If we consider the case of viral targets, there are several reasons for discrepancies in activity results, IC_50_ vs EC_50_ values, between assays conducted on isolated enzymes and cellular systems. These include, among others, differences in the assay protocols (e.g., virus strain, construct, incubation time, temperature, buffer composition, etc.) and in the complexity of the microenvironment of the protein target (e.g., DENV2 protease enzyme is located near the endoplasmic reticulum in a replication complex that involves multiple interactions with other viral and host proteins), effects originating from pharmacokinetic-related factors (e.g., membrane permeability, metabolic stability) or even pharmacodynamic-related factors linked to the distribution of the protein conformational populations of the studied target in the cellular environment. The apparent EC_50_ values of compounds in viral replication assays are prone to variations depending on the cell type and virus strain used, the cell passage number, the virus input or multiplicity of infection, the time of addition of the assessed compound, and the readout of the assay.^20^

#### B) The Choice of Reference Compounds to Assess Assay Compatibility

When an assay is conducted using different procedures, the results of the assessed ligands do not vary consistently, thus altering the relative ranking of hits. Distinct assay protocols may reflect specific mechanisms of action, and these insights will be lost, if mixed data are combined in a single AI/ML model. As mentioned earlier, in the antiviral cellular assays for many enveloped viruses, as an example, the assessment of the inhibitory effect on the virus binding/attachment step is done by incubation of the compound with the virus inoculum and the host cells at 4 °C. However, an incubation at 37 °C is used to study inhibition of the entry/fusion step, which depends on protein and membrane dynamics.^17^

Given the variable sensitivity of compounds, the selection of references for checking the compatibility of assays should be done carefully. For instance, from a mechanistic perspective, the IC_50_ of a noncompetitive inhibitor can give an incorrect indication of the compatibility between assays that are conducted under distinct substrate-related parameters. On a more complex level, conformational changes in a protein, in response to pH, salinity, or other factors, could be confined to specific residues or binding areas. Accordingly, the effects on ligands depend on their recognition requirements and the interactions governing their binding affinity and inhibitory potency. For instance, both compounds A and B in Figure 1 are competitive inhibitors of DENV protease. The limited sensitivity of the dipeptide boronic acid to changes in the binding site may be due to the strength of the interaction of the electrophilic boronic acid warhead, which anchors the ligand to the protein. On the other hand, the benzyloxyphenylglycine residue in inhibitors B and C can lead to distinct binding modes depending on the space distribution in specific subpockets in the active site.^11,21^ The IC_50_ of inhibitor A varies by less than 2-fold for condition 3 vs 5, while inhibitor B exhibits a significant change by 68-fold for its IC_50_, and 117-fold for its *K*_i_. If we compare conditions 4 and 5, the enzyme concentration used is 100 nM, the substrate, Bz-nKRR-AMC, has a Michaelis constant (*K*_m_) of 90 μM and 75 μM, for conditions 4 and 5, respectively. The enzyme activity parameters show maximum velocity of the enzymatic reaction (*V*_max_) of 0.0108 μM/s and 0.0014 μM/s, in addition to turnover number (*K*_cat_) of 0.0827 s^–1^ and 0.0143 s^–1^, for conditions 4 and 5, respectively (for more information about the substrate and enzyme activity parameters under the assessed conditions, the reader can refer to the Supporting Information of reference 7). Despite these values, the inhibitory activity of compound B was significantly lower for condition 5 than for condition 4. For inhibitor C, the *K*_d_ varies by 23-fold for condition 3 vs 6. In the binding assay, condition 6 is assessed outside the pH range for the established catalytic activity of this enzyme. Notably, the experimental data to correlate the changes in the medium (pH, salts, buffers, etc.) to alterations in the protein conformation are still limited, because there are not many examples where these factors were varied and experimental structures have been obtained.

It is useful to use more than one reference compound that can serve as a probe for different binding areas on the target. As shown recently, it was possible to retain just 1% of publicly accessible compound potency data after applying an extensive curation strategy in comparing assays.^1^ Introducing further restrictions to ensure a match between more assay-related details will limit the ability to obtain large data sets from the literature for protein targets exhibiting ligand-independent conformational plasticity, as discussed for the case of enzymes. This is due to the fact that these targets will present binding site heterogeneity depending on the medium in which the assay is performed (i.e, pH, salts, buffers, and others).

## Assay Data Handling Considerations

Experimental studies that screen compound groups or libraries will increase the quantity of available data. Furthermore, studies that enrich target-specific knowledge are equally important in improving the data quality for model training and development. With respect to handling assay-related data, simpler ML approaches would be straightforward to apply and less data-demanding;^22^ however, they are not designed to handle higher levels of data complexity. Classical ML algorithms rely on “hand-engineered” features (selected by the data scientist or developed tools) in the form of molecular descriptors, which allow easy interpretability of model outcomes, i.e., how features affect the predicted activity. DL utilizes neural networks (NNs) with an architecture composed of multiple processing layers that automatically learn relevant features from the data.^23^ DL methods are typically data-intensive and require substantial computing power and advanced AI expertise. Furthermore, these methods are comparably opaque and may not provide a rationale for specific explanations or predictions.^24^

Although DL requires more training data than classical ML models, it offers an advantage through transfer learning, where knowledge from an already trained model is leveraged to improve performance on a related task with scarce data.^25^ This concept underpins the breakthrough of large language models (LLMs), which integrate and apply knowledge from diverse domains and disciplines, supporting flexible reasoning and contextual understanding. GenAI, as a subset of the broader DL domain, has a creative ability to generate novel chemical entities and to explore uncharted territories of the chemical space.^26^ Nevertheless, high-end GenAI models have the limitation of being even more data-hungry than DL or ML, and consequently, training and operating these models require significant computational resources. Additionally, they are more error-prone, and model evaluation metrics remain an area in need of further refinement to assess the novelty, druglikeness, and synthetic accessibility of the generated compounds.^24,26^

An interesting development is the use of LLM-driven agentic AI systems to support scientific research goals. In the specific case study presented in this article, ML can serve as a starting point, especially when the number of compounds is limited, provided that heterogeneous experimental conditions are avoided. DL would be more appropriate for a larger number of compounds per experimental setup, and when there is a need to integrate data from more than one assay condition, representing distinct conformations or targets. This can take place through multistage models (Figure 2), where individual models can be clustered together such that each one acts as a proxy or surrogate for a distinct conformation or conformational ensemble, or even for a different target protein. GenAI holds a unique advantage in generating compound suggestions with specific activity against a particular assay condition or with broader effects spanning all clustered models.

**Figure 2:**
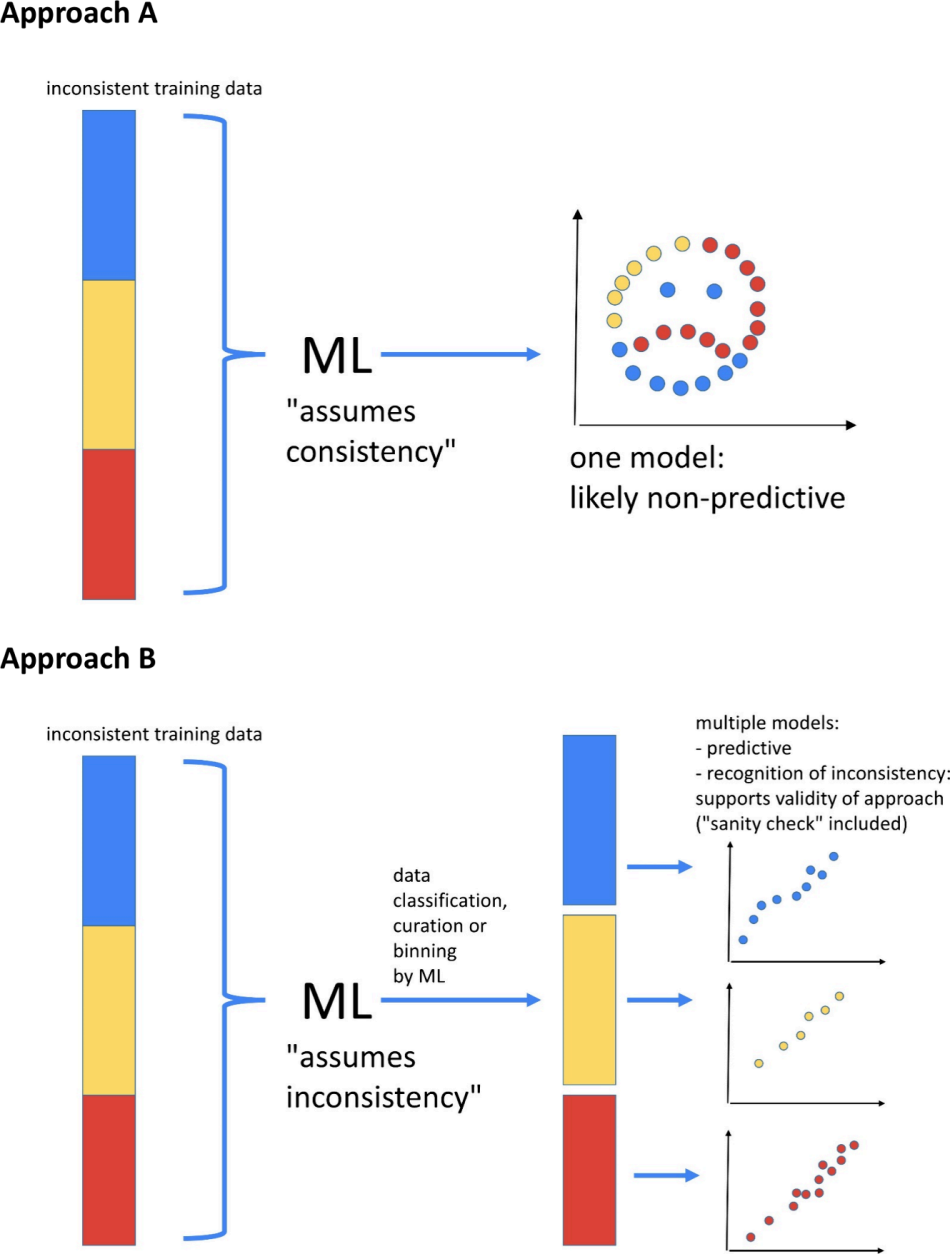
Illustration showing the importance of sorting the data fed into ML models to improve the model predictivity. Two approaches are depicted: A) An approach that assumes the consistency of the training data, causing model confusion. In this case, the heterogeneous data are fed into a single model, which likely lacks predictivity. B) An approach that assumes the inconsistency of the training data, and results in predictive models. The data heterogeneity is addressed by curation or sorting into multiple individual models that are clustered together. ML is used as an umbrella term for DL and non-DL/NNs approaches.

Overall, we consider that the combined expertise in computer engineering and drug discovery can certainly unlock vast opportunities in the field. In structural biology, for example, the DL-based AlphaFold2 and AlphaFold3 (developed by DeepMind, a subsidiary of Google, and highlighted by the Nobel prize in 2024 for AlphaFold2) have a remarkably positive impact by enabling accurate protein structure prediction.^27,28^ From this perspective, we decided to request the feedback of a researcher in the field of AI/ML on a number of questions, which are, in our opinion, of relevance to the drug discovery community (Figure 3).

**Figure 3:**
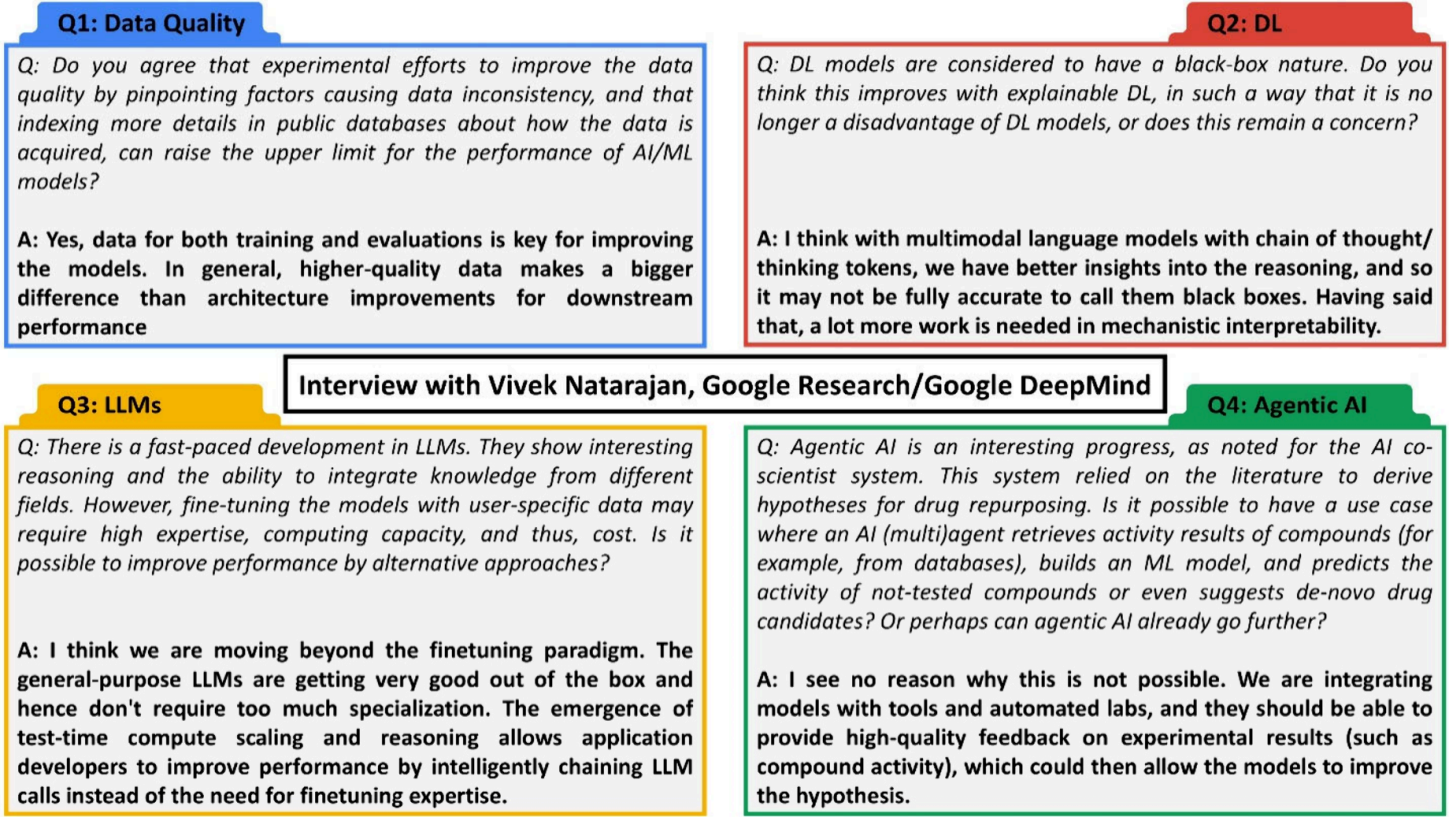
Questions to Vivek Natarajan, Google DeepMind, and his answers. Vivek Natarajan recently co-led the development of the AI co-scientist,^29^ a multiagent AI system built with Gemini 2.0. This virtual scientific collaborator aims to support scientific research, and was assessed for biomedical applications such as drug repurposing.

## Computational Studies of Protein–Ligand Interactions for Targets with Conformational Plasticity

The variations addressed in earlier sections, regarding the results of experimental binding and inhibition assays, are expected to take place as well in the predicted protein–ligand interactions by computational approaches. Comparability of the outcomes of these studies depends on considering the similarity of the settings used to generate the results. The main challenge in this area is the current lack of accurate free-energy scoring functions, either physics-based or ML-based. Current docking or free energy calculations are still limited in terms of predictions beyond lead optimization, e.g. when new chemical entities are designed or screened in a de novo approach. Molecular dynamics (MD) simulations have several limitations when studying protein targets exhibiting high plasticity, such as, the semiempirical nature of force fields, which have several approximations, and the insufficient simulation time to capture large conformational changes. Validation against experimental data to select the most relevant conformation(s) for virtual screening campaigns is hindered by the lack of ground-truth in free energy predictions. Recently, progress in sampling alternate conformational states of a protein was reported by integrating an in-solution experimental method, small-angle scattering (SAS), with AlphaFold.^30^ The complementarity between AI/ML and experimental techniques can be particularly promising in gaining insights about the conformational landscape of a target protein and its influence on protein–ligand interactions. However, in general, we need predictive models that go beyond the “one conformational solution”, mostly presented by AlphaFold and other current DL-based models, and can predict ensembles of conformations consistent with statistical mechanics to estimate alternate protein–ligand interactions.

## Conclusion

In conclusion, careful comparison of assay-related information before combining bioactivity results from different literature sources or databases is important to obtain scientifically useful outcomes, particularly in the case of highly dynamic protein targets. Topic-focused studies can provide guidance for selecting relevant assay procedures, and reference compounds that can serve as sensitive points of comparison between different protocols. These curation steps are essential to improve the quality of the combined data, but are expected to restrict their size. Generating target-specific consistent testing results by screening compound libraries under relevant conditions allows for extended data sets. This approach is expected to substantially raise the upper limit for the performance of AI/ML models. Leveraging advances in the AI/ML field can further improve the analysis and interpretation of assay results. While the detailed discussion in this Viewpoint was limited to enzymes and viral surface proteins, as model examples, similar issues may apply to other targets, e.g. receptors, as well. However, it is important to consider additional factors, which are specifically relevant to these targets, e.g. the expression level in the case of receptors.

## Abbreviations

AI: Artificial intelligence
BLI: Biolayer interferometry
β-OG: n-octyl-β-d-glucoside
DENV: Dengue virus
DL: Deep learning
E protein: Envelope protein
EC_50_: Half-maximal effective concentration
IC_50_: Half-maximal inhibitory concentration
ITC: Isothermal titration calorimetry
*K*_cat_: Turnover number
*K*_d_: Dissociation constant
*K*_i_: Inhibition constant
*K*_I_: Inactivation constant
*K*_inact_: Maximal rate of covalent inactivation
*K*_m_: Michaelis constant
LLMs: Large language models
MD: Molecular dynamics
ML: Machine learning
NNs: Neural networks
SARS-CoV-2 M^pro^: Severe acute respiratory syndrome coronavirus 2 main protease
SAS: Small-angle scattering
SPR: Surface plasmon resonance
*V*_max_: Maximum velocity of the enzymatic reaction
Boceprevir: (1R,2S,5S)-N-(4-amino-1-cyclobutyl-3,4-dioxobutan-2-yl)-3-((S)-2-(3-(*tert*-butyl)­ureido)-3,3-dimethylbutanoyl)-6,6-dimethyl-3-azabicyclo­[3.1.0]­hexane-2-carboxamide;
Compound A: ((R)-1-((S)-2-benzamido-5-guanidinopentanamido)-4-guanidinobutyl)­boronic acid;
Compound B: N-((S)-1-(((S)-6-amino-1-(((R)-2-amino-1-(4-(benzyloxy)­phenyl)-2-oxoethyl)­amino)-1-oxohexan-2-yl)­amino)-5-guanidino-1-oxopentan-2-yl)­benzamide;
Compound C: N-((S)-1-(((S)-6-amino-1-(((R)-2-amino-1-(4-((3-methoxybenzyl)­oxy)­phenyl)-2-oxoethyl)­amino)-1-oxohexan-2-yl)­amino)-5-guanidino-1-oxopentan-2-yl)-[2,2′-bithiophene]-5-carboxamide;
Abz-VVTLQSGDap­(Dnp)­R-OH: ((S)-2-(2-((S)-2-((S)-5-amino-2-((S)-2-((2S,3S)-2-((S)-2-((S)-2-(2-aminobenzamido)-3-methylbutanamido)-3-methylbutanamido)-3-hydroxybutanamido)-4-methylpentanamido)-5-oxopentanamido)-3-hydroxypropanamido)­acetamido)-3-(4-hydroxy-3-nitrophenyl)­propanoyl)-l-arginine;
Bz-nKRR-AMC: N-((6S,9S,12S,15S)-1-amino-12-(4-aminobutyl)-9-(3-guanidinopropyl)-1-imino-6-((4-methyl-2-oxo-2H-chromen-7-yl)­carbamoyl)-8,11,14-trioxo-2,7,10,13-tetraazanonadecan-15-yl)­benzamide.
